# Drug resistance in breast cancer is based on the mechanism of exocrine non-coding RNA

**DOI:** 10.1007/s12672-024-00993-3

**Published:** 2024-05-01

**Authors:** Simin Ye, Shiyu Chen, Xiaoyan Yang, Xiaoyong Lei

**Affiliations:** 1 School of Pharmaceutical Science, Hengyang Medical College, University of South China, 28 Western Changsheng Road, Hengyang, 421001 Hunan People’s Republic of China; 2 The Hunan Provincial Key Laboratory of Tumor Microenvironment Responsive Drug Research, University of South China, 28 Western Changsheng Road, Hengyang, 421001 Hunan People’s Republic of China

**Keywords:** Breast cancer, Exosomes, Non-coding RNAs, Drug resistance

## Abstract

Breast cancer (BC) ranks first among female malignant tumors and involves hormonal changes and genetic as well as environmental risk factors. In recent years, with the improvement of medical treatment, a variety of therapeutic approaches for breast cancer have emerged and have strengthened to accommodate molecular diversity. However, the primary way to improve the effective treatment of breast cancer patients is to overcome treatment resistance. Recent studies have provided insights into the mechanisms of resistance to exosome effects in BC. Exosomes are membrane-bound vesicles secreted by both healthy and malignant cells that facilitate intercellular communication. Specifically, exosomes released by tumor cells transport their contents to recipient cells, altering their properties and promoting oncogenic components, ultimately resulting in drug resistance. As important coordinators, non-coding RNAs (ncRNAs) are involved in this process and are aberrantly expressed in various human cancers. Exosome-derived ncRNAs, including microRNAs (miRNAs), long-noncoding RNAs (lncRNAs), and circular RNAs (circRNAs), have emerged as crucial components in understanding drug resistance in breast cancer. This review provides insights into the mechanism of exosome-derived ncRNAs in breast cancer drug resistance, thereby suggesting new strategies for the treatment of BC.

## Introduction

Cancer is a devastating disease that poses a significant threat to human life and health worldwide. Breast cancer (BC) is one of the most common types of cancer and continues to be the focus of intensive research [1–3]. Studies have shown that breast cancer is responsible for approximately 2.26 million deaths in 2020, making it the primary cause of high mortality among women [4, 5]. Breast cancer exhibits considerable morphological and molecular heterogeneity and can be categorized into several subtypes, including Luminal A, Luminal B, HER2-positive, triple-negative, and basal-like [6]. Currently, treatment options for breast cancer include surgery, radiotherapy, chemotherapy, immunotherapy, and targeted therapy [7]. Chemotherapy is one of the primary treatments for tumors and plays an important role in controlling tumor progression. Of course, long-term use of chemotherapeutic agents is often accompanied by the development of drug resistance, which further leads to treatment failure. Therefore, the mechanism of tumor drug resistance and drug resistance prevention or reversal strategies have been a hot issue in tumor therapy research. Mechanisms of drug resistance in breast cancer patients include alterations in drug transporters, changes in target molecules, and activation of survival pathways. Therefore, understanding these mechanisms of drug resistance in breast cancer is crucial for developing new treatments and improving patient outcomes.

Exosomes are small extracellular vesicles encapsulated by a lipid bilayer, ranging in size from 40 to 100 nm. Early endosomes are formed by the plasma membrane and subsequently develop into late endosomes, which can eventually give rise to multivesicular bodies (MVBs). These MVBs partly release exosomes through cytosolic exocytosis and partly fuse with lysosomes for material degradation [8–10]. Recent studies have highlighted the involvement of exosomes in drug resistance, demonstrating that exosomes released by drug-resistant cancer cells can induce drug resistance in other cells [11]. In breast cancer, exosomes play a crucial role in drug resistance due to their diverse cargo, which includes various types of RNA (messenger RNAs (mRNAs), microRNAs (miRNAs), long non-coding RNAs (lncRNAs), and circular RNAs (circRNAs)), DNA, proteins, lipids, and metabolites [12–14]. Importantly, the levels of exosomes in the sera of breast cancer patients are often higher compared to those in healthy individuals [15, 16].

Although a significant amount of current research focuses on studying drug resistance in exosomes, the lack of a detailed systematic summary of exosome-derived RNAs in the process of resistance induction has led to unclear mechanisms of action of various drugs in the treatment of breast cancer [17, 18]. A comprehensive systematic review is necessary to elucidate further the action mechanism of the significant components of exosomes in generating drug resistance. This review covers three main areas. Firstly, it describes the mechanisms of drug resistance in breast cancer and the biogenesis and composition of exosomes. Secondly, it investigates the role of exosome-derived miRNAs, lncRNAs, and circRNAs in breast cancer drug resistance. Thirdly, it delves into the role of exosome-derived ncRNAs in breast cancer cross-resistance. Finally, it provides a comprehensive summary that highlights the significance of exosome-derived ncRNAs in addressing chemotherapy resistance in breast cancer treatment. In conclusion, this review highlights the critical role of exosome-derived ncRNAs in the development of drug resistance in breast cancer. Additionally, it underscores the significance of further research in enhancing cancer treatment outcomes. This will offer up-to-date insights and theoretical guidance for the studying drug resistance in breast cancer.

## Mechanisms of drug resistance in breast cancer

Chemotherapy has always played an important role in the comprehensive treatment of breast cancer. The main chemotherapeutic agents currently approved are tamoxifen, doxorubicin, trastuzumab, paclitaxel and cisplatin, among others. Initially breast cancer is sensitive to chemotherapy, however, over time, breast cancer cells can employ a variety of mechanisms to develop resistance [19]. These mechanisms mainly include drug efflux and inactivation, activation of bypass signaling or survival pathways, induction of epithelial-mesenchymal transition (EMT) and stem cell-like properties [7]. Breast cancer resistance protein (BCRP or ABCG2) is involved in cancer multidrug resistance and transports anticancer drugs out of cells [20]. Alterations in the estrogen receptor pathway (e. g., ESR1 mutations) or upstream growth factor signaling pathways (e. g., PI3K/Akt/mTOR pathway) can cause drug resistance in breast cancer patients [21]. Activation of EMT can also induce breast cancer cells to acquire resistance to chemotherapy [22]. A very small number of drug-resistant cells are present in breast cancer, and this population exhibits stem cell-like properties and high tumorigenic potential [23].

## The source and internal composition of exosomes

It has been found that various type of cells secrete exosomes, with mutant cells secrete more exosomes compared to healthy cells [24]. Exosomes play a significant role in various processes associated with tumorigenesis and cancer progression, such as the reconstruction of the tumor microenvironment, immunosuppression, angiogenesis, metastasis, and chemoresistance [25, 26]. Exosomes act as mediators of intercellular communication, capable of delivering and exchanging nucleic acids, proteins and lipids to activate or inhibit different signaling pathways in target cells [27].

**Fig. 1:**
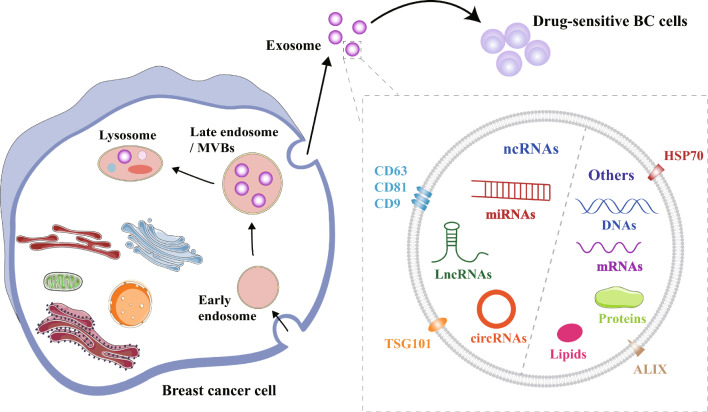
The biogenesis and composition of exosomes. Early endosomes are formed through the inward outgrowth of the plasma membrane and then mature into late endosomes, which eventually form multivesicular bodies. Some multivesicular bodies fuse with lysosomes to undergo material degradation, while others are released into the extracellular environment through cytosolic exocytosis. Exosomes contain a variety of substances such as various types of RNA (mRNAs, miRNAs, lncRNAs, and circRNAs), DNA, proteins, lipids, and metabolites. Notable biomarkers found in exosomes include members of the tetraspanin family (CD63, CD81, and CD9), ALIX, TSG101, and HSP70

Exosomes are identified using key markers such as the tetraspanin family (CD63, CD81, and CD9), ALIX, TSG101, and HSP70 (Fig. 1) [28]. These transmembrane proteins have diverse roles in biological processes including cell adhesion, membrane fusion, motility, invasion, and exosome regulation [29]. Recent studies have highlighted the crucial role of exosomes in cancer development and their potential for breast cancer treatment. Importantly, the contents of exosomes can reflect the characteristics and condition of the cells that synthesize them, and are resistant to degradation by extracellular proteases. Furthermore, exosomes can be readily detected in various biological fluids and their cargo composition can be accurately determined by liquid biopsy. As a result, exosomes are increasingly recognized as a new diagnostic and prognostic biomarker for malignant tumors, including breast cancer [30].

## Exosomal miRNAs and drug resistance in breast cancer

Non-coding RNAs (ncRNAs) have received significant attention as important regulators among the various components of exosomes. Abnormal expression of ncRNAs has been identified in numerous human cancers and has been associated with the regulation of gene expression [31]. Moreover, studies have demonstrated that exosomes derived from breast cancer exert a critical role in drug resistance by containing various ncRNAs such as miRNAs, lncRNAs, and circRNAs. This phenomenon stands as the leading cause of cancer cells acquiring multidrug resistance, offering a promising target for cancer therapy [2, 32].

It is known that exosomes can enhance the survival of breast cancer cells in the presence of drug therapy through three mechanisms: drug neutralization, drug efflux, and immune system suppression [17]. Studies have demonstrated that HER2-positive breast cancer cells secrete exosomes containing high levels of activated HER2, and these exosomes can directly bind to trastuzumab, a drug that targets HER2, thereby reducing the efficacy of the treatment [33]. Additionally, exosomes have the ability to encapsulate drugs and expel them from cancer cells. Previous studies have suggested that exosomes can trap doxorubicin within cancer cells and then eliminate it [34]. Furthermore, exosomes can transfer miRNAs from drug-resistant cells to non-resistant cells, thus contributing to drug resistance [17]. However, the molecular mechanism and signaling pathway through which exosomes promote drug resistance in breast cancer cells is complex and not yet fully elucidated. In the following sections, we discuss this phenomenon with respect to different drugs.

### Tamoxifen resistance

Approximately 70% to 75% of breast cancers highly express estrogen receptor alpha (ERα), and most ERα-positive breast cancers are dependent on estrogen signaling, which initially results in a favorable response to endocrine therapy [35]. Tamoxifen (TMX), an estrogen receptor antagonist, is a commonly utilized and highly efficacious endocrine therapy for the treatment of ERα-positive breast cancer [36]. By competitively binding to the estrogen receptor, it can reduce the potential invasiveness and associated mortality of breast cancer. Despite its initial success, about 30% of breast cancer patients undergoing tamoxifen therapy will experience recurrence, and the exact mechanism behind this resistance remains unclear [37]. Recent reports suggest that abnormalities in exosome-derived miRNA may contribute to tamoxifen resistance. Therefore, it is crucial to understand the mechanisms underlying drug resistance to develop effective solutions for the treatment of breast cancer.

Research has demonstrated that exosomes secreted by tamoxifen-resistant MCF-7 cells are capable of inducing drug resistance in tamoxifen-sensitive cells [38]. Zhao et al*.* conducted a study in which they found that exosomes derived from tamoxifen-resistant cells were internalized by recipient breast cancer cells, leading to the promotion of drug resistance and inhibition of apoptosis in the recipient cells due to the presence of miRNA-205 in the exosomes. Subsequent research showed that miRNA-205, present in the exosomes, directly silenced the E2F transcription factor 1 (E2F1) target gene, thereby promoting tamoxifen resistance and tumorigenesis in breast cancer [39]. These findings suggest that miRNA-205 may serve as a promising biomarker for drug resistance in tumor treatment. In another study, Liu et al. found that miR-9-5p inhibited gene expression of ADIPOQ (Adiponectin, C1Q and Collagen Domain Containing) through exosomal transport by bioinformatics analysis and luciferase activity assay and ultimately played an important role in tamoxifen resistance of breast cancer cells. Further in vivo experiments in nude mice confirmed that tumors injected with exosomal miR-9-5p showed improved resistance to tamoxifen [40]. Similarly, exosomes derived from tamoxifen-resistant cells carried miR-221 and miR-222, which negatively targeted and regulated P27 and ERα genes, converting tamoxifen-sensitive cells into tamoxifen-resistant cells [41]. The transfer of exosome-derived miR-221 and miR-222 plays a crucial role in the communication of tamoxifen resistance, representing the first evidence that these miRNAs act as signaling molecules that mediate the spread of drug resistance in breast cancer. This discovery may provide a novel therapeutic strategy to overcome tamoxifen resistance in ER-positive breast cancer cells by enhancing cellular drug sensitivity through modulating the delivery of tumor-promoting or tumor-suppressing exosomal miRNAs. Furthermore, miR-181a-2 in exosomes derived from tamoxifen-resistant cells downregulated ERα expression, induces the transmission of tamoxifen resistance, and prolonged irreversible blockade by estrogen signaling [42]. This finding sheds light on the role of exosomal miRNAs in targeting the estrogen receptor mechanism during the metastasis of drug-resistant phenotypes in breast cancer cells. These studies suggest that exosome-mediated miRNA transfer can modulate target genes and affect tamoxifen resistance in breast cancer, indicating that targeting miRNAs as potential therapeutic targets to address the problem of tamoxifen resistance.

### Doxorubicin resistance

Doxorubicin (DOX) is a commonly used first-line anticancer drug that functions as a DNA topoisomerase II inhibitor. Its cytotoxic properties are mediated through the induction of DNA strand breaks or disruptions in RNA metabolism [43]. Despite the advantages of doxorubicin, such as increased survival rates in breast cancer patients, the development of drug resistance is almost inevitable. Resistance to DOX has become a significant barrier to successful oncological treatment.

Exosomes containing miRNAs derived from drug-resistant breast cancer cells have the ability to traverse the tumor microenvironment and transmit drug resistance to neighboring sensitive cells by transferring specific miRNAs, which in turn alter gene expression within the sensitive cells [44]. For example, the resistance to doxorubicin can be transmitted from resistant MCF-7 cells to normal MCF-7 cells through the exosome-mediated transfer of miR-222 [45]. However, the exact mechanism of this transfer is still unknown. Through microarray analysis, 52 novel miRNAs with elevated expression levels in drug-resistant cells were identified. Bioinformatics studies of these miRNAs could help overcome chemoresistance in future breast cancer treatments [46]. Subsequent research revealed that doxorubicin-resistant MCF-7 cells release exosomes carrying specific miRNAs (miR-100, miR-222, and miR-30a), which regulate the distribution of the cell cycle during intercellular transfer, decrease apoptosis induced by drug treatment, and confer doxorubicin resistance to sensitive MCF-7 cells [47]. Moreover, miR-770 present in tumor-derived exosomes can be transferred to macrophages. This transfer leads to the regulation of DNA repair and the activation of M1 polarization, ultimately inhibiting chemoresistance in breast cancer cells. MiR-770 acts as a potential tumor suppressor by targeting STMN1 to downregulate it. This regulation of STMN1 in turn affects apoptosis, epithelial-mesenchymal transition, metastasis, and chemoresistance to doxorubicin in triple-negative breast cancer, thereby altering the tumor microenvironment [48]. Therefore, miR-770 may serve as a new prognostic marker and provide new insights to understand the underlying mechanisms of chemotherapy resistance and metastasis. Furthermore, recent studies have shown that the exosome miR-181b-5p secreted by DOX-resistant cells can be taken up by recipient cells to induce DOX resistance by down-regulating BCLAF1 expression and subsequent p53/p21-mediated evasion of senescence [49]. This suggests that the development of inhibitors targeting miR-181b-5p has therapeutic potential in the treatment of DOX-resistant BC patients.

### Trastuzumab resistance

Compared to other types of breast cancer, HER2-positive breast cancer exhibits enhanced proliferative, invasive, and metastatic capabilities. Therapeutic drugs that specifically target HER2 receptors have demonstrated more favorable treatment outcomes [50]. Trastuzumab is one such drug, which is a monoclonal antibody that obstructs the extracellular structural domain of HER2. It is extensively utilized for the treatment of both early-stage and metastatic breast cancer in cases of HER2 overexpression [51]. Nevertheless, the effectiveness of trastuzumab in treating HER2-positive breast cancer has been challenged due to the development of acquired resistance [52].

Han et al. conducted a bioinformatics analysis and identified miR-567 as a potential player in trastuzumab resistance, with lower levels of miR-567 in trastuzumab-resistant cells compared to parental cells. In this study, exosome-derived miR-567 targeted autophagy-associated protein 5 (ATG5) and post-transcriptionally regulated its expression, thereby inhibiting autophagy. As a result, this process enhanced the drug sensitivity of breast cancer cells to trastuzumab [53]. The study emphasizes the significant role of miR-567 in the regulating of autophagy and its potential to overcome trastuzumab resistance in breast cancer cells. Additionally, identifying predictors of trastuzumab resistance is critical for the developing of effective treatment options. In HER2-positive breast cancer patients, miR-1246 and miR-155 have been identified as predictive and prognostic factors for trastuzumab resistance [54]. Conducting further research on the mechanism underlying miR-1246 and miR-155-induced trastuzumab resistance would facilitate the development of novel drugs that can be combined with trastuzumab to enhance treatment outcomes for HER2-positive breast cancer.

### Paclitaxel resistance

Paclitaxel (PTX) is a potent chemotherapeutic agent that is commonly used as a first-line treatment for various types of cancer. Its mechanism of action involves disrupting the normal polymerization and disassembly of microtubules in the bloodstream. Specifically, PTX binds to microtubules and stabilizes them, leading to cell cycle arrest and subsequent apoptosis in dividing cells [55]. In the case of advanced breast cancer, anthracycline/paclitaxel-based chemotherapy regimens are frequently employed as neoadjuvant chemotherapy to shrink tumors and prevent metastasis. However, despite its effectiveness, PTX resistance remains a significant obstacle in the treatment of metastatic breast cancer, resulting in low survival rates for patients. Therefore, it is crucial to understanding the underlying mechanisms of PTX resistance in breast cancer cells to develop effective therapies for breast cancer [56].

In their study, Yang et al*.* made a significant discovery that chemotherapy treatment triggers the EZH2/STAT3 pathway in tumor cells. This activation leads to the binding of miR-378a-3p and miR-378d to promoter regions. Subsequently, these two miRNAs are taken up by cancer cells that survive chemotherapy, resulting in the development of resistance to paclitaxel in breast cancer. The resistance is caused by the direct targeting of Wnt antagonist DKK3 and the Notch inhibitor NUMB by the miRNAs. As a result, the Wnt and Notch stem cell pathways are activated [57]. Furthermore, miR-155 has been identified as playing a crucial role in promoting cell proliferation, angiogenesis, invasion, and recurrence, as well as accelerating tumor growth. Therefore, understanding the mechanisms associated with miR-155 in cancer is of significant clinical importance [58]. Research has also demonstrated that exosomes secreted by paclitaxel-resistant breast cancer cells can transfer miR-155, thereby promoting chemoresistance in breast cancer cells [59]. Consequently, targeting the miR-155 signaling pathway may prove to be an effective therapeutic strategy.

### Cisplatin resistance

Cisplatin, a widely employed and potent medication, is utilized for the treatment of various solid malignant tumors [60]. Its mechanism of action is centered around disrupting DNA repair mechanisms through binding with purine bases on DNA, thereby resulting in DNA damage and activation of multiple signal transduction pathways. These pathways then induce either necrosis or apoptosis of cancer cells [61]. Nevertheless, the development of drug resistance poses a substantial challenge in the effective utilization of cisplatin and often culminates in unsuccessful treatment outcomes [62].

Recent research has provided evidence that MDA-MB-231 breast cancer cells, which exhibit resistance to cisplatin, are capable of transmitting resistance information to other breast cancer cell lines, including MCF-7, SKBR-3, and cisplatin-sensitive MDA-MB-231 breast cancer cells. This transfer of resistance occurs through the release of exosomes that contain miR-423-5p [63]. This study contributes to our comprehension of the mechanisms underlying cisplatin resistance in breast cancer, and suggests that targeting miR-423-5p could serve as a potential strategy for overcoming drug resistance in the treatment of triple-negative breast cancer.

miRNAs play a crucial role in various cellular processes, such as cell cycle development, cell differentiation, and cancer development. Their impact on physiological and cellular processes has been associated with cancer development [64]. Additionally, the transfer of miRNAs through exosomes serves as an essential molecular mechanism for intercellular communication. The study specifically examines the involvement of exosome-derived miRNAs in drug resistance in breast cancer (Table 1).

**Table 1:** Exosome-derived miRNAs studies in breast cancer drug resistance

miRNA	Target/ Pathway	Drug resistance	References
miRNA-205	E2F1	Tamoxifen	[39]
miR-9-5p	ADIPOQ	Tamoxifen	[40]
miR-221/222	P27, ERα	Tamoxifen	[41]
miR-181a-2	ERα	Tamoxifen	[42]
miR-100, miR-222, miR-30a	regulate the cell cycle distribution	Doxorubicin	[47]
miR-770	STMN1	Doxorubicin	[48]
miR-181b-5p	BCLAF1	Doxorubicin	[49]
miR-567	ATG5	Trastuzumab	[53]
miR-1246, miR-155	not determined	Trastuzumab	[54]
miR-378a-3p, miR-378d	EZH2/STAT3	Paclitaxel	[57]
miR-155	not determined	Paclitaxel	[59]
miR-423-5p	not determined	Cisplatin	[63]

## Exosomal LncRNAs and drug resistance in breast cancer

In recent years, research has revealed that lncRNAs are important participants in various cellular processes, including proliferation, apoptosis, cell migration, and invasion [65–67]. These RNA molecules, which exceed 200 nucleotides in length and lack protein-coding ability, have also been identified as critical regulators of pathways related to cancer [68, 69]. Specifically, they play crucial roles in epigenetic regulation by interacting with DNA regulatory elements or chromatin-modifying complexes [70, 71]. Furthermore, emerging evidence suggests that lncRNAs are involved in breast cancer drug resistance, underscoring their potential as therapeutic targets for cancer treatment (Table 2).

**Table 2:** Exosome-derived LncRNAs studies in breast cancer drug resistance

LncRNA	Mechanism of action	Drug resistance	References
lncRNA UCA1	AKT/mTOR signaling pathway	Tamoxifen	[72]
lncRNA H19	not determined	Doxorubicin	[74]
lncRNA AGAP2-AS1	not determined	Trastuzumab	[75]
lncRNA Linc00969	HUR	Trastuzumab	[76]
lncRNA NEAT1	miR-133b/CXCL12	Paclitaxel	[79]

### Tamoxifen resistance

Exosomes have been found to transfer lncRNA UCA1 from drug-resistant cells to drug-sensitive cells in breast cancer, leading to the development of resistance to tamoxifen. It is hypothesized that this transfer involves subsequent regulation of the AKT/mTOR signaling pathway [72]. In addition, there is a correlation between high expression levels of lncRNA HOTAIR and the adverse effects of tamoxifen treatment in breast cancer patients, as evidenced by its presence in serum exosomes [73]. The lack of validated biomarkers that can indicate prognosis and chemotherapy resistance in breast cancer is a significant factor contributing to disease progression. This highlights the potential of lncRNA HOTAIR in serum exosomes as a diagnostic and prognostic biomarker, fulfilling an important clinical need.

### Doxorubicin resistance

Numerous studies have examined the role of lncRNA H19 as an oncogenic lncRNA in breast cancer progression. However, its contribution to drug resistance in breast cancer has been relatively neglected. Han et al. found that lncRNA H19 mediates doxorubicin resistance in breast cancer cells through exosomal transfer [74]. Despite this significant finding, further investigation is necessary to determine the precise mechanism underlying this process.

### Trastuzumab resistance

RNA-binding protein hnRNPA2B1 played a critical role in sorting and loading lncRNA AGAP2-AS1 into exosomes, and highly expressed lncRNA AGAP2-AS1 could convey resistance to trastuzumab in breast cancer cells [75]. This reveals that lncRNA AGAP2-AS1 can be used as a therapeutic target and help discover new treatment strategies for HER2-positive breast cancer patients. Liu et al*.* found that exosome-derived lncRNA Linc00969 could induce trastuzumab resistance in breast cancer by binding to HUR to increase HER-2 protein expression and enhance HER-2 mRNA stability [76].

## Exosomal circRNAs and breast cancer drug resistance

As a newly discovered class of ncRNAs, circRNAs have been found to be involved in a variety of biological processes, including cell proliferation, migration, gene expression, and regulation of certain responses. Recent research has emphasized the critical role of circRNAs in the regulation of cancer, as their abnormal expression has been associated with cancer progression, drug resistance, and prognosis. Therefore, circRNAs present a promising avenue for the identification of potential biomarkers in cancer research [77]. However, our current understanding of the involvement of tumor-derived exosome circRNAs in drug resistance remains limited, as only a few studies have been conducted to date (Table 3).

**Table 3:** Exosome-derived CircRNAs studies in breast cancer drug resistance

CircRNA	Mechanism of action	Drug resistance	References
CircRNA-CREIT	PKR/eIF2α signaling pathway	Doxorubicin	[78]
circ_UBE2D2	MiR-200a-3p	Tamoxifen	[80]
circ-MMP11	miR-153-3p/ ANLN	Lapatinib	[81]
circBACH1	miR-217/G3BP2	Paclitaxel	[82]

The second stem loop of circRNA-CREIT played a critical role in facilitating the binding to the double-stranded RNA binding motifs (dsRBMs) of the PKR protein. By serving as a scaffold, circRNA-CREIT enhanced the interaction between PKR and the E3 ligase HACE1, thereby promoting the proteasome-dependent degradation of PKR proteins by modulating the ubiquitination of K48 linkages. As a result, the PKR/eIF2α signaling pathway was inhibited, which is essential for activating the RACK1/MTK1 stress-responsive apoptotic signaling pathway by triggering the assembly of stress granules (SG). The study findings suggest that a combination of ISRIB (a small molecule inhibitor of SG) and doxorubicin can effectively inhibit tumor growth in TNBC. Furthermore, circRNA-CREIT can reduce doxorubicin resistance in breast cancer cells through exosomal delivery, highlighting its potential as a promising diagnostic biomarker for breast cancer [78]. Therefore, targeting circRNA-CREIT and SGs could represent an effective therapeutic strategy for combating chemoresistance in TNBC.

## RNA–RNA interactions

**Fig. 2:**
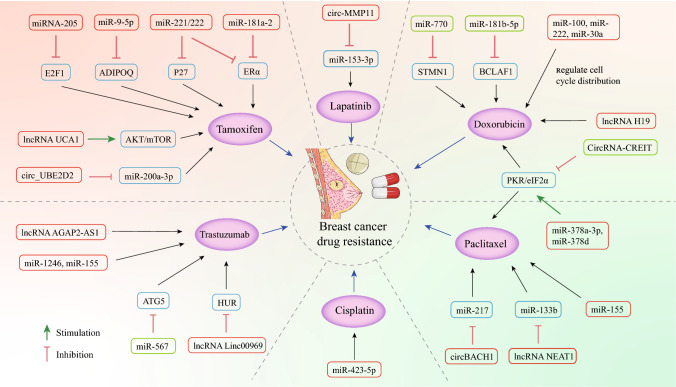
The drug resistance mechanisms of tumor-derived exosomal miRNAs, lncRNAs, and circRNAs in breast cancer. Tumor-derived exosomal ncRNAs can transfer drug resistance characteristics from drug-resistant cells to drug-sensitive cells

MiRNAs bind to their target mRNAs through MRE (miRNA Response Element) sequences, which are also present in lncRNAs and circRNAs. When these MRE-containing lncRNAs or circRNAs competitively bind to miRNAs, it leads to an increase in the level of miRNA-regulated mRNA transcription [71]. The exosome-derived lncRNA NEAT1 upregulated CXCL12 expression through competitively binding to miR-133b, promoted breast cancer cell migration and growth, and induced paclitaxel resistance in breast cancer cells [79]. In their study, Hu et al*.* demonstrated that the transfer of circ_UBE2D2 via exosomes enhances tamoxifen resistance in estrogen receptor-positive breast cancer. This effect is mediated by the interaction between circ_UBE2D2 and miR-200a-3p, which in turn regulates cell viability, metastasis, and ERα levels in vivo [80]. Exosome-derived circ-MMP11 acted as a sponge for miR-153-3p to regulate ANLN expression, thereby promoting resistance to lapatinib in breast cancer cells. This provides a therapeutic target for breast cancer treatment [81]. Furthermore, paclitaxel-induced exosome-derived circBACH1 promotes paclitaxel resistance in recipient breast cancer cells by upregulating G3BP2 expression through sponging miR-217, this upregulation increases cell survival and promotes cell stemness [82]. These findings present a novel therapeutic target for chemoresistance and metastasis in breast cancer (Fig. 2).

## Prospects and outlook

Despite the significant progress made in recent years, there remain several limitations and challenges in fully understanding the complex role of exosomal ncRNAs in drug resistance associated with breast cancer. Therefore, further exploration is imperative to elucidate the resistance mechanisms of key exosomal ncRNAs and to devise novel and more efficacious therapeutic strategies. Additionally, it is crucial to exploit tumor-derived exosomal ncRNAs as diagnostic and therapeutic targets to improve the clinical outcomes for breast cancer patients. One promising approach is to inhibit the secretion of exosomes derived from tumor cells. Another approach involves the development of exosomes carrying therapeutic agents, such as functional proteins, ncRNAs, and various chemotherapeutic agents for targeted delivery to tumor cells. Investigating the utilization of exosomes as a delivery vehicle for breast cancer therapy is a potential area of research that could significantly enhance our understanding of their behavior. Overall, continued research on tumor-derived exosomal ncRNAs will undoubtedly exert a positive impact on clinical outcomes for breast cancer patients.

## Conclusion

In conclusion, exosomes derived from breast cancer contain various ncRNAs, including miRNAs, lncRNAs, and circRNAs. Abnormal regulation of these ncRNAs has been linked to breast cancer progression, with the promotion of tumor cell proliferation, invasion, metastasis, and cachexia. This review aims to elucidate the mechanisms by which exosome-derived ncRNAs contribute to chemoresistance in cancer cells. Drug resistance poses a significant challenge in the treatment of breast cancer, and its underlying mechanisms are complex. Exosomes, which contain ncRNAs, serve as essential mediators of intercellular communication and play a vital role in breast cancer progression. A growing body of research has linked an increasing number of ncRNAs to drug resistance in breast cancer. Tumor cells release exosomes containing specific miRNAs, lncRNAs, and circRNAs, which are then transported to adjacent or distant non-resistant receptors. This transfer of exosomes enables previously drug-sensitive cells to acquire drug resistance, highlighting the importance of identifying their contents and transfer between cells. Consequently, the insights gained from this study can aid in the development of a theoretical framework and potential therapeutic targets for overcoming cancer drug resistance in a clinical setting.

## Data Availability

No data was used for the research described in the article.
